# Supplementary material for: Identification of Food Spoilage Fungi Using MALDI-TOF MS: Spectral Database Development and Application to Species Complex
Source: J Fungi (Basel). 2024 Jun 28;10(7):456. doi: 10.3390/jof10070456 (PMC11277938; doi:10.3390/jof10070456)
Supplement: Supplementary file 1 [file jof-10-00456-s001.zip › jof-3062193-supplementary.pdf]

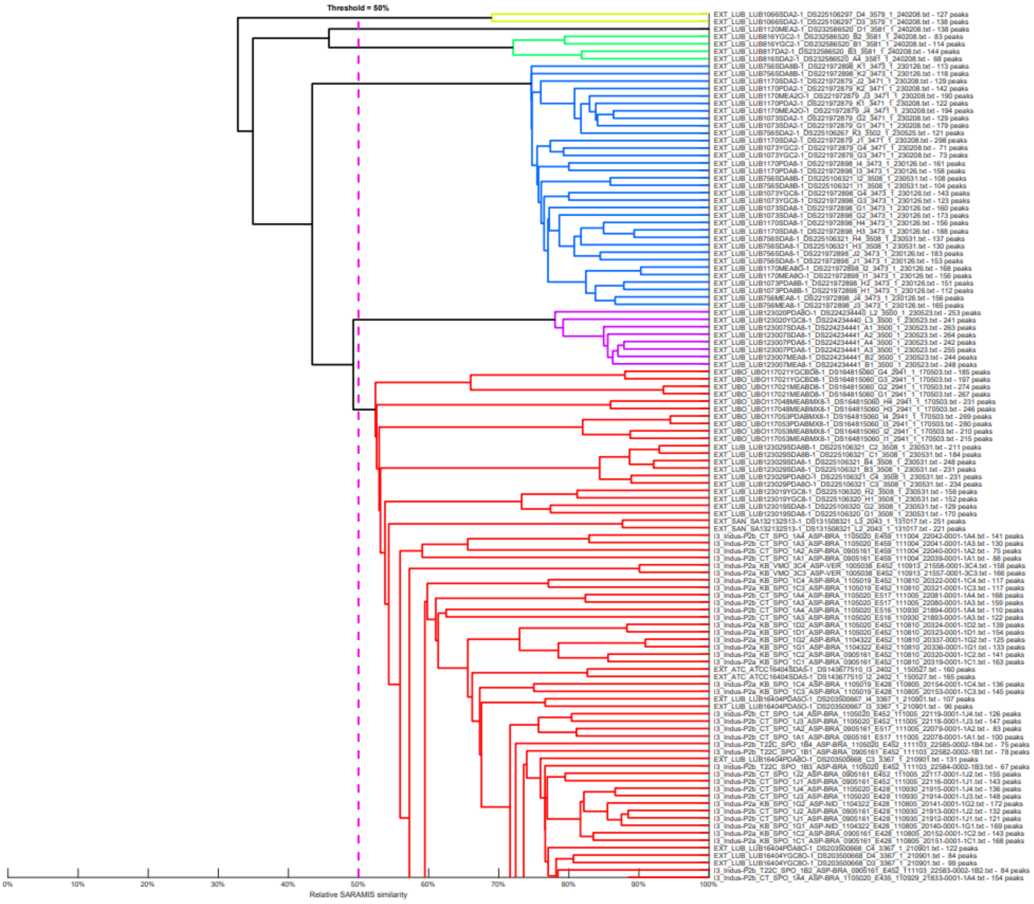
Threshold = 50%
0%
10%
20%
30%
40%
50%
60%
70%
80%
90%
100%
Relative SARAMIS similarity

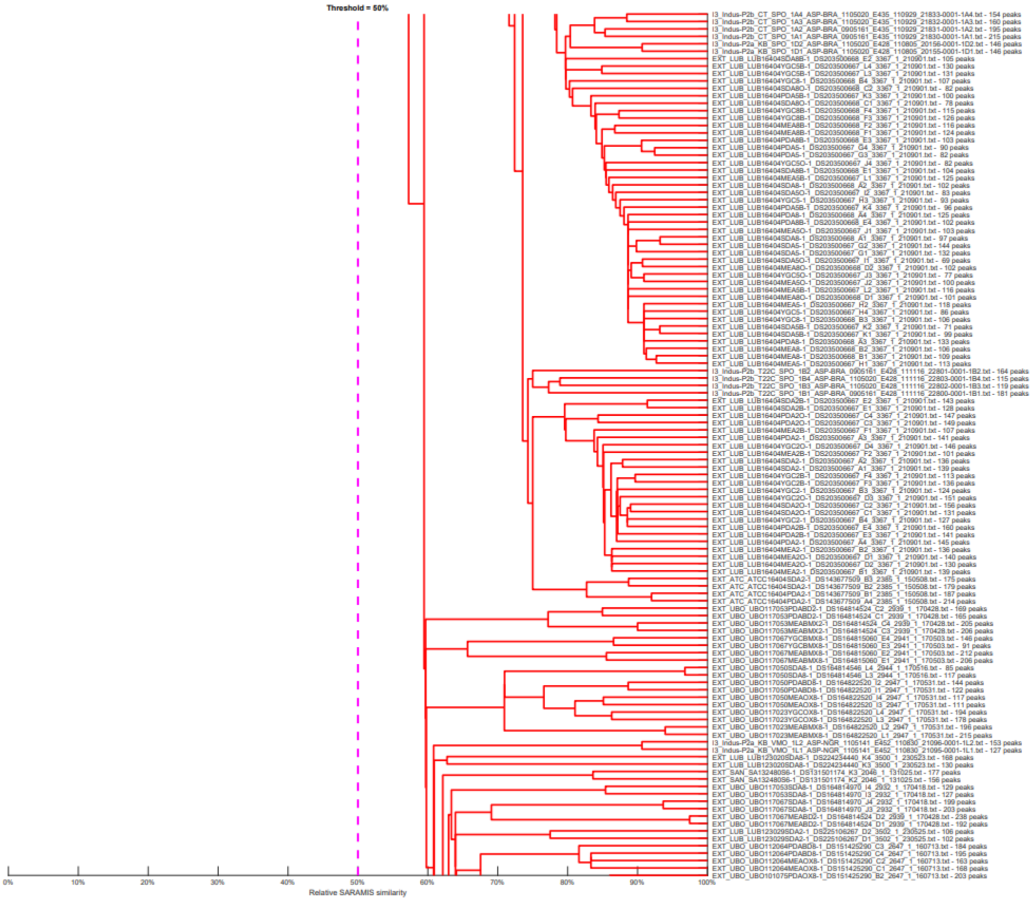
Threshold = 50%
0%
10%
20%
30%
40%
50%
60%
70%
80%
90%
100%
Relative SARAMIS similarity

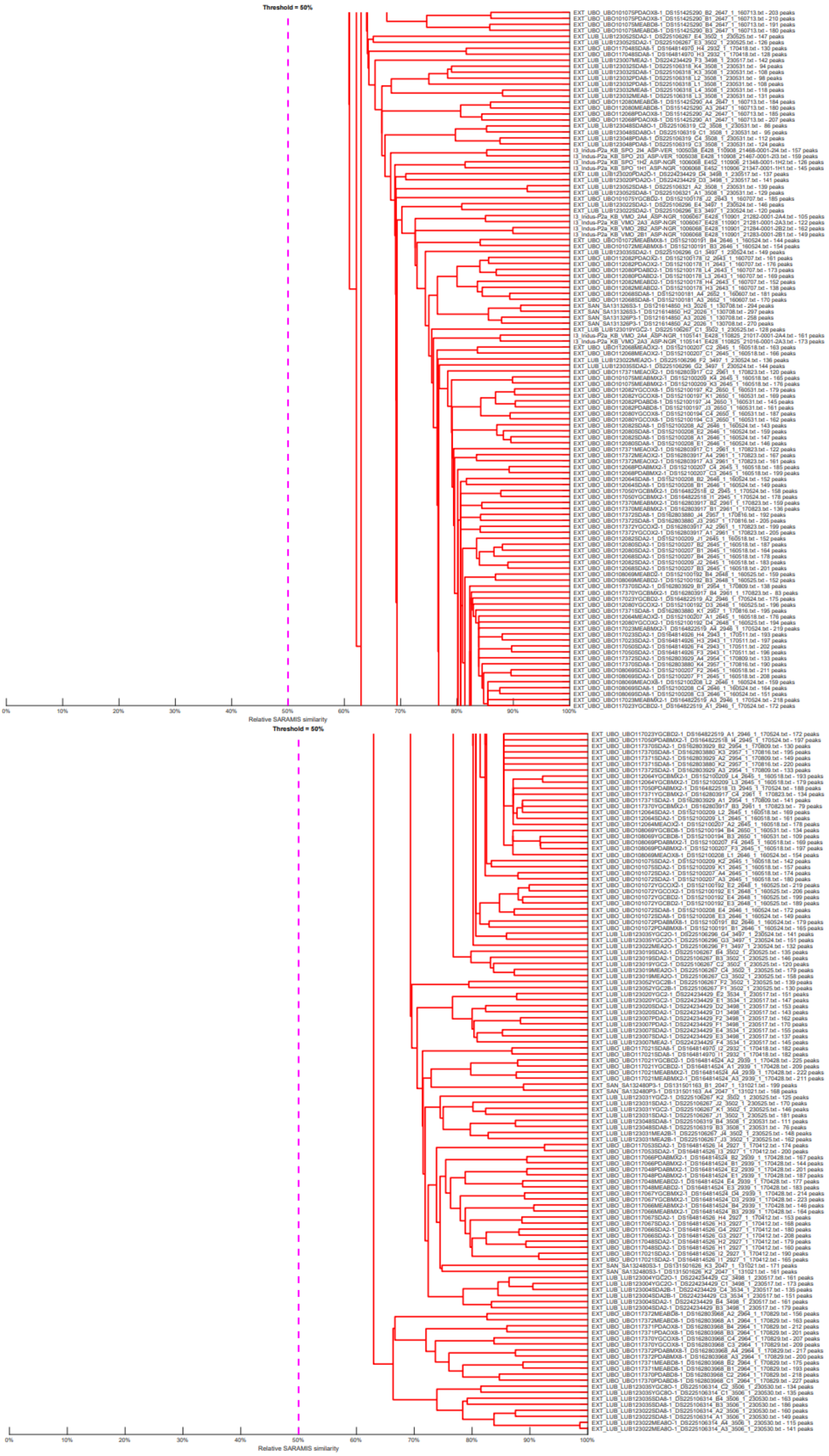

Figure S1. Dendrogram based on spectral similarity between the different strains of *Aspergillus* series *nigri* included in the VITEK MS ® Knowledge Base

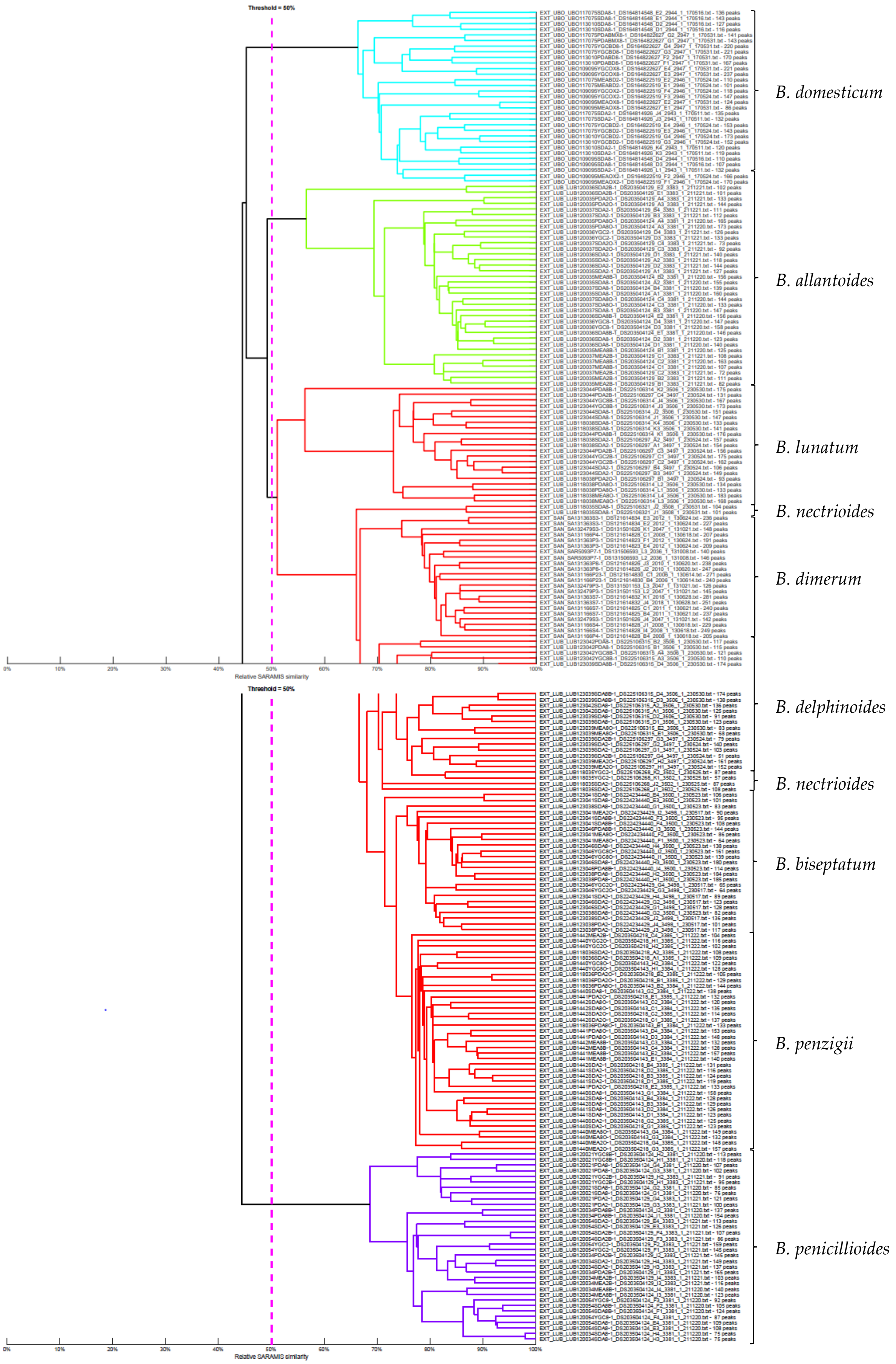

Figure S2. Dendrogram based on spectral similarity between the different strains of *Bisifusarium dimerum* complex included in the VITEK MS ® Knowledge Base

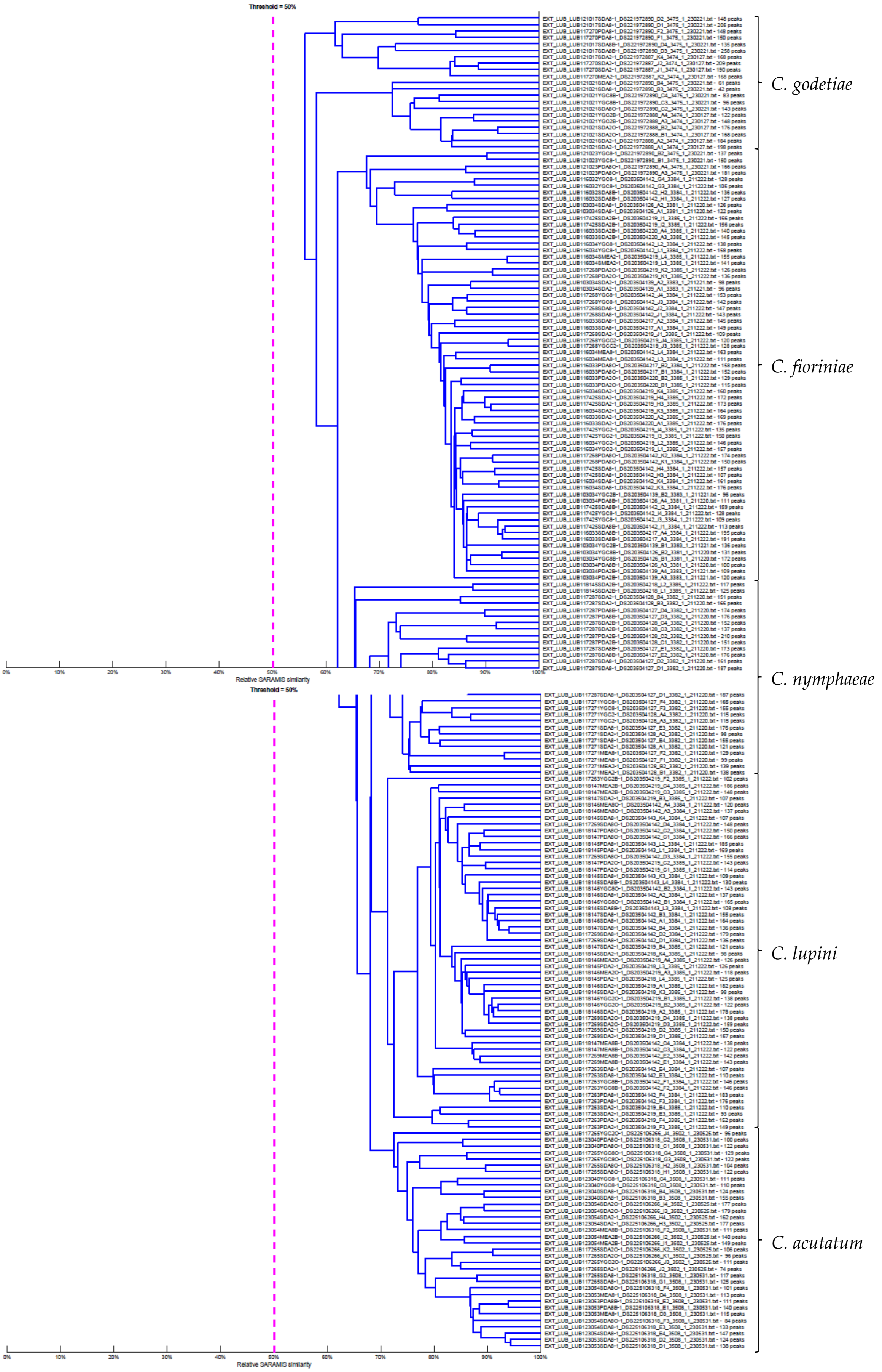

Figure S3. Dendrogram based on spectral similarity between the different strains of *Colletotrichum acutatum* complex included in the VITEK MS ® Knowledge Base

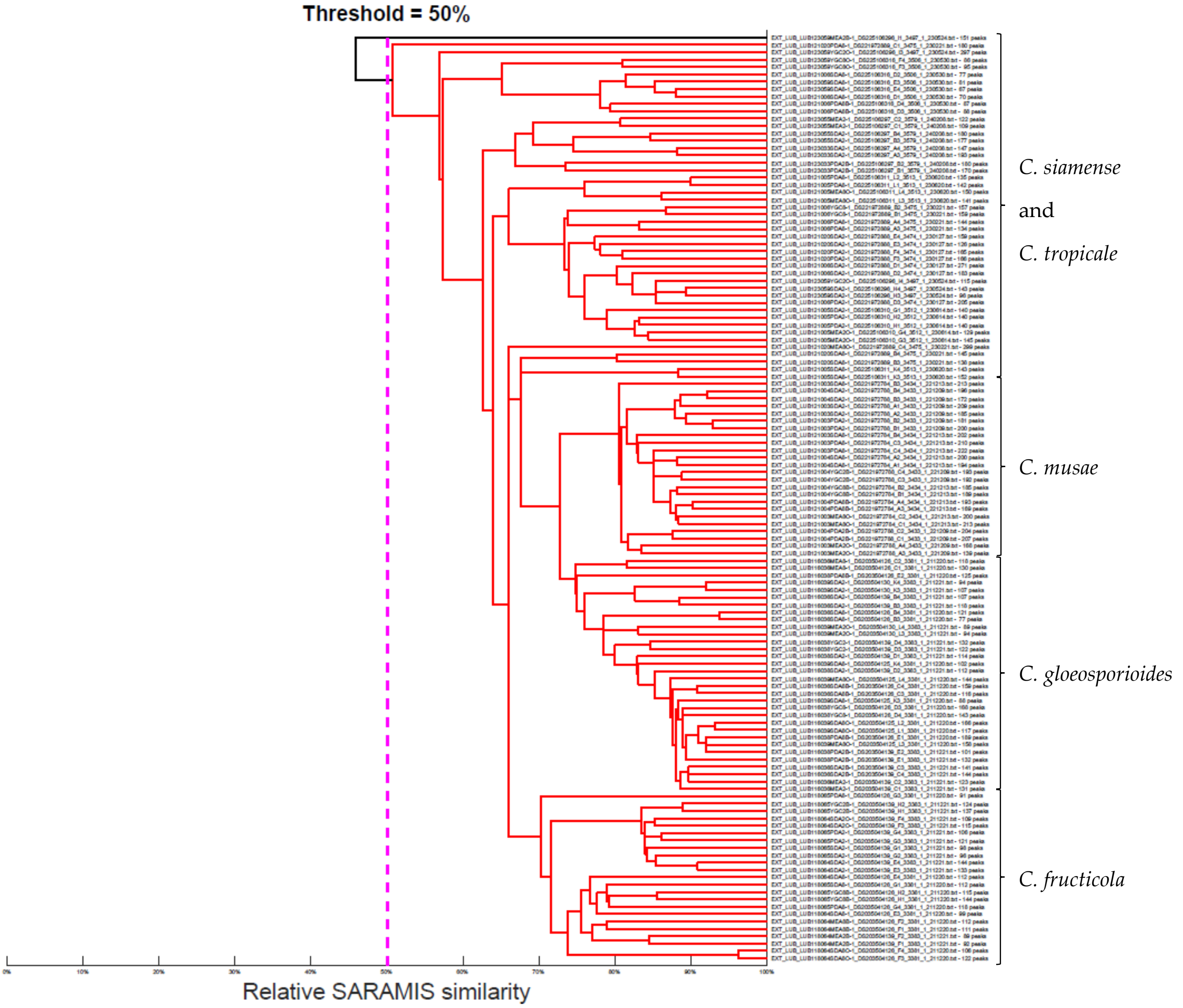

Figure S4. Dendrogram based on spectral similarity between the different strains of *Colletotrichum gloeosporioides* complex included in the VITEK MS ® Knowledge Base

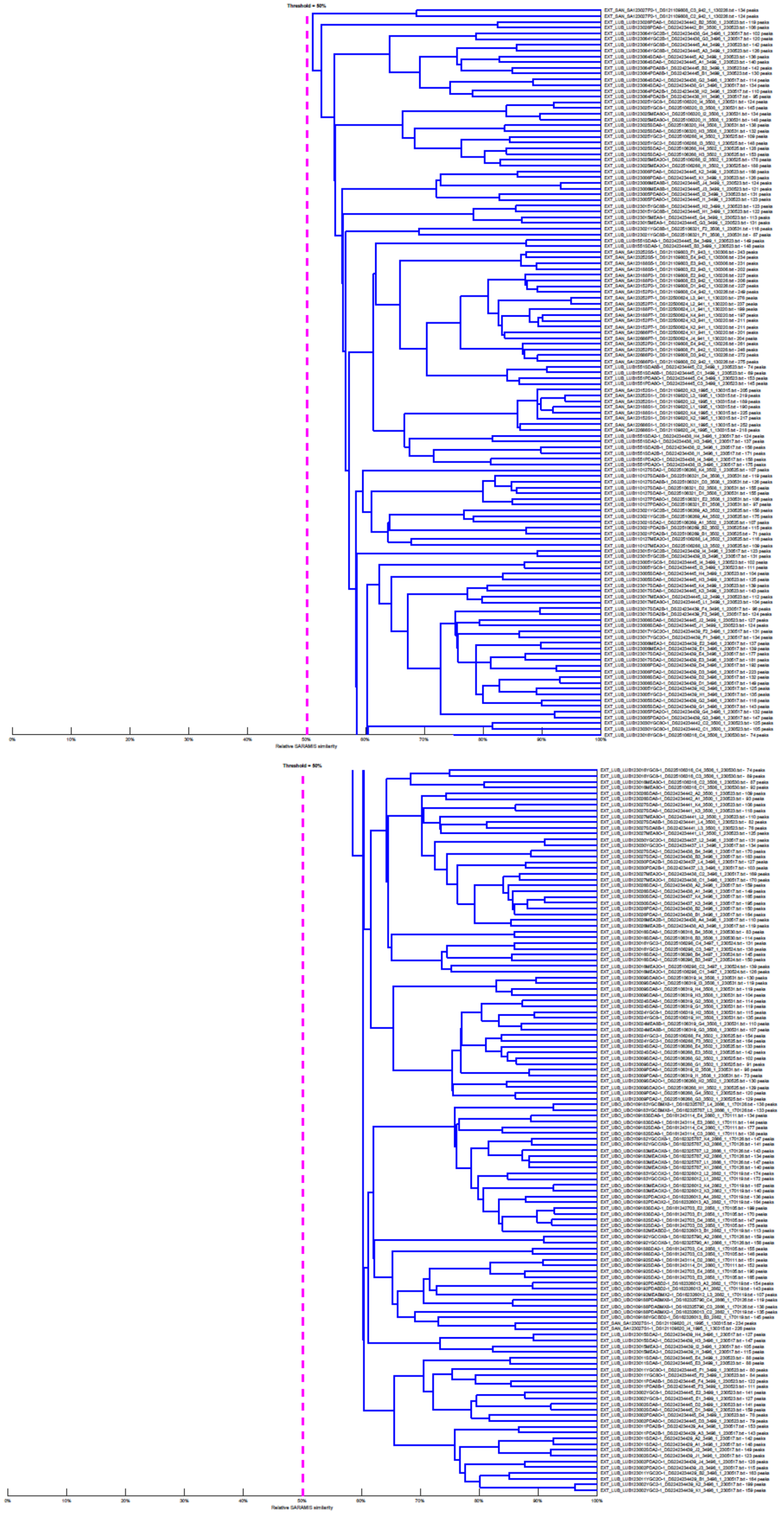

Figure S5. Dendrogram based on spectral similarity between the different strains of *Mucor circinelloides* complex included in the VITEK MS ® Knowledge Base
